# Changes in Oxidative and Nitrosative Stress Indicators and Vascular Endothelial Growth Factor After Maximum-Intensity Exercise Assessing Aerobic Capacity in Males With Type 1 Diabetes Mellitus

**DOI:** 10.3389/fphys.2021.672403

**Published:** 2021-08-06

**Authors:** Łukasz Tota, Bartłomiej Matejko, Małgorzata Morawska-Tota, Wanda Pilch, Sandra Mrozińska, Tomasz Pałka, Tomasz Klupa, Maciej T. Malecki

**Affiliations:** ^1^Department of Physiology and Biochemistry, University of Physical Education in Krakow, Krakow, Poland; ^2^Department of Metabolic Diseases, Jagiellonian University Medical College, Krakow, Poland; ^3^University Hospital in Krakow, Krakow, Poland; ^4^Department of Sports Medicine and Human Nutrition, University of Physical Education in Krakow, Krakow, Poland; ^5^Institute of Basic Research, Department of Chemistry and Biochemistry, University of Physical Education in Krakow, Krakow, Poland

**Keywords:** type 1 diabetes, oxidative stress, graded exercise, exercise capacity, muscle physiology

## Abstract

In type 1 diabetes mellitus (T1DM), chronic hyperglycemia causes reactive oxygen and nitrogen species production. Exercise alters the oxidant-antioxidant balance. We evaluated the aerobic capacity and oxidant-antioxidant balance changes after maximum-intensity exercise in T1DM patients. The study involved 30 T1DM participants and 23 controls. The patients’ average age was 23.4 ± 5.1 years, with a body mass index of 24.3 ± 3.1 kg m^–2^ and with satisfactory glycemic control. Among the controls, the respective values equaled 24.7 ± 2.9 years and 22.9 ± 2.1 kg m^–2^. Aerobic capacity was assessed with a treadmill test. Peak minute oxygen uptake was significantly lower in T1DM compared with the controls (44.7 ± 5.7 vs. 56.0 ± 7.3 mL kg^–1^ min^–1^). The total oxidant capacity measured by total oxidative status/total oxidative capacity (TOS/TOC) equaled 321.5 ± 151 μmol L^–1^ before and 380.1 ± 153 μmol L^–1^ after exercise in T1DM, and 164.1 ± 75 and 216.6 ± 75 μmol L^–1^ in the controls (*p* < 0.05 for all comparisons). A significant difference in the ratio of total antioxidant status/total antioxidant capacity (TAS/TAC) between the groups after the treadmill test was observed (*p* < 0.05). Nitrosative stress indicators where significantly higher in the T1DM group both before and after the exercise. In conclusion, diabetic patients demonstrated a lower aerobic capacity. The TOS/TOC and nitrosative stress indicators were significantly higher in T1DM before and after the test.

## Introduction

Chronic hyperglycemia is the main cause of diabetic complications. In patients with diabetes, the production of reactive oxygen and nitrogen species (RONS) is intensified by biochemical pathways associated with hyperglycemia, such as through glucose autoxidation, non-enzymatic glycation of proteins, mitochondrial overproduction of RONS, overproduction and activation of kinase C, xanthine oxidation, as well as the intensification of aldol and polyol reductase pathways (Martín-Gallán et al., 2007). Scientific literature supports the hypothesis that the overproduction of RONS during chronic hyperglycemia results in a lower activity of glutathione and of the key enzymes with antioxidant properties, such as superoxide dismutase and glutathione peroxidase (Picu et al., 2017).

Despite the high toxicity of free radicals, a small amount of reactive oxygen and nitrogen species is necessary for proper cell function; however, their excessive production or insufficient antioxidant defense of the human body may lead to intracellular damage. Free radicals react with the molecules that comprise the basic cell structure, such as proteins, lipids, and nucleic acids, and distort their biological function (Frijhoff et al., 2015). This phenomenon is referred to as oxidative stress (Frijhoff et al., 2015; Ighodaro, 2018). Its occurrence in diabetes results from an increase in the level of free radicals, as well as disturbances in the activity of endogenous enzymatic antioxidants and a decrease in the concentration of low-molecular-weight antioxidants (Brownlee, 2001).

Regular physical activity, through adaptation mechanisms, increases antioxidant capacity which should have a beneficial effect in patients with type 1 diabetes mellitus (T1DM) (Narendran et al., 2020).

However, among patients with diabetes who already have high free radical levels, a further increase in RONS takes place during any high-intensity exercise, causing concern that the intensification of oxidative and nitrosative stress will be significant (Noble and Shen, 2012).

The occurrence of nitrosative stress results from a significant intensification of the reaction between nitric oxide and superoxide anion radical, which leads to the formation of peroxynitrite, a potent oxidant (Estévez and Jordán, 2002). In turn, peroxynitrite is an effective oxidant and nitrating agent whose presence in the cell results in decreased concentrations of intracellular antioxidants and damage to proteins, lipids, and nucleic acids. Nitric oxide, a universal signaling molecule, is continuously produced by three isoforms of nitric oxide synthase (Förstermann and Sessa, 2012).

Nitration of tyrosine residues in proteins affects their structure and function; it can alter the catalytic activity of enzymes, in extreme cases causing their inactivation (Pacher et al., 2007). Nitrotyrosine, a marker of nitrosative stress, has been identified in diseases with an inflammatory background, including those associated with diabetes; this indicates an increased formation of reactive nitrogen species during these diseases (Kowluru et al., 2004; Ihnat et al., 2007).

Advances in treatment options, as well as modern monitoring methods and improved markers of glycemia have resulted in more effective glycemic control. Despite this, patients with diabetes may develop vascular complications, such as retinopathy. A significant role in its occurrence is played by growth factors, including vascular endothelial growth factor (VEGF), growth hormone, and transforming growth factor β. VEGF production is enhanced in diabetic retinopathy, probably in response to hypoxia (Keenan et al., 2007). There are a number of substances that stimulate VEGF secretion; these include interleukins, growth factors, hormones, nitric oxide, and reactive oxygen species (Ihnat et al., 2007).

During intense exercise, substantial changes are observed in the dynamic state of oxidant-antioxidant balance, which constitutes a cellular defense system neutralizing the effects of pro-oxidants (Powers et al., 2016). The formation of free radicals during physical activity depends on the type, intensity, duration, and frequency of exercise, as well as the individual antioxidant potential (Tofas et al., 2020). Very intense physical training can contribute to oxidative stress even in professional athletes (Tofas et al., 2020). Among type 2 diabetes patients, a positive correlation was observed between the percentage of total body fat and the total oxidant status (TOS) [TOS/total oxidant capacity (TOC)] (Picu et al., 2017). A positive correlation was also revealed between TOS/TOC and glycated hemoglobin (HbA1c) concentrations, as well as between TOS/TOC and glycemic levels (Picu et al., 2017). Of note, total antioxidant status/total antioxidant capacity (TAS/TAC), measuring plasma antioxidant capacity, represents an alternative marker reflecting the redox state in the body in a more effective manner than the evaluation of a single circulating antioxidant (Erel, 2005).

As a result of the introduction of new methods of insulin therapy, T1DM is no longer a barrier to intense physical activity or even extreme sports training (Vlahek et al., 2013). The continuous education on the influence of physical activity on the level of glycemia and the risk of hypoglycemia should be a concern not only among physicians and patients but also among sports instructors and trainers working with patients with diabetes (Gawrecki et al., 2012).

In a long-term perspective study, regular physical activity in the form of endurance training had a beneficial effect on the cardiovascular system function, lipid profile, and carbohydrate metabolism. It also helped maintain a stable energy balance and normalize body mass in both healthy individuals and T1DM patients (Absil et al., 2019). However, early direct physiological and biochemical reactions to physical exercise in T1DM patients have not yet been properly examined. New research on the oxidant-antioxidant balance shift in T1DM patients, which is a consequence of maximum-intensity exercise, would allow us to better understand the biochemical reactions in this disease (Réus et al., 2019).

In the present study, we aimed to assess aerobic capacity and analyze how oxidative and nitrosative stress induced by maximum-intensity exercise would affect endothelial function in patients with T1DM as compared with healthy participants with similar body mass index (BMI) and age.

## Materials and Methods

### Participant Characteristics

Our outpatient clinic is a tertiary diabetes center and T1DM patients are almost exclusively treated with personal insulin pumps. The number of T1DM patients on multiple daily injections under our care is very limited. This study involved 53 young male participants: 30 T1DM patients and 23 controls, all being university students from Krakow. All T1DM individuals were treated with a personal insulin pump, a method representative of the majority of T1DM patients in our center. All participants were considered to be otherwise healthy and did not receive any medication other than insulin. They were aged 23.4 ± 5.1 years on average, with a mean BMI of 24.3 ± 3.1 kg m^–2^. In the control group, the mean age was 24.7 ± 2.9 years and the mean BMI equaled 22.9 ± 2.1 kg m^–2^. The control group participants had a negative history of diabetes and of elevated glucose levels. The diabetic group inclusion and exclusion criteria are presented in Table 1.

**TABLE 1:** The T1DM group inclusion and exclusion criteria.

Inclusion criteria	Exclusion criteria
– Diagnosis of T1DM – Current treatment with a personal insulin pump – Most recent HbA1c level ≤ 9%	– Chronic complications of diabetes: non-proliferative retinopathy; proliferative retinopathy; diabetic maculopathy; autonomic neuropathy and polyneuropathy; diabetic nephropathy of stages III–V: chronic kidney disease – Severe hypoglycemia or diabetic ketoacidosis within the previous 7 days – Significant cardiovascular, respiratory, or locomotor system disorder – Glycemia before a standardized meal > 250 mg dL^–1^ (11.1 mmol L^–1^) or < 60 mg dL^–1^ (3.3 mmol L^–1^), or < 70 mg dL^–1^ (3.8 mmol L^–1^) with accompanying hypoglycemic symptoms – Postprandial glycemia after 90 min > 300 mg dL^–1^ (13.8 mmol L^–1^) and the presence of ketone bodies in urine – Body mass index ≥ 30 kg m^–2^ – Abnormalities in the resting electrocardiography – Lack of qualification by a physician

*T1DM, type 1 diabetes mellitus; HbA1c, glycated hemoglobin; severe hypoglycemia was defined by altered mental and/or physical functioning that required the assistance of another person for recovery (American Diabetes Association, 2019.*

The control group included healthy men qualified by a physician to perform maximum-intensity exercise, with an age and BMI matched to those in the T1DM group. Owing to the open label nature of the experiment, sham infusion was not used in the control group.

The study followed the tenets of the Declaration of Helsinki and was approved by the Jagiellonian University Bioethics Committee (approval No. 1072.6120.113.2017 of September 28, 2017). The patients were informed about the aims and protocol of the study and provided their written consent to participate. The exercise tests were performed under a constant supervision of a physician.

### Study Protocol

**FIGURE 1:**
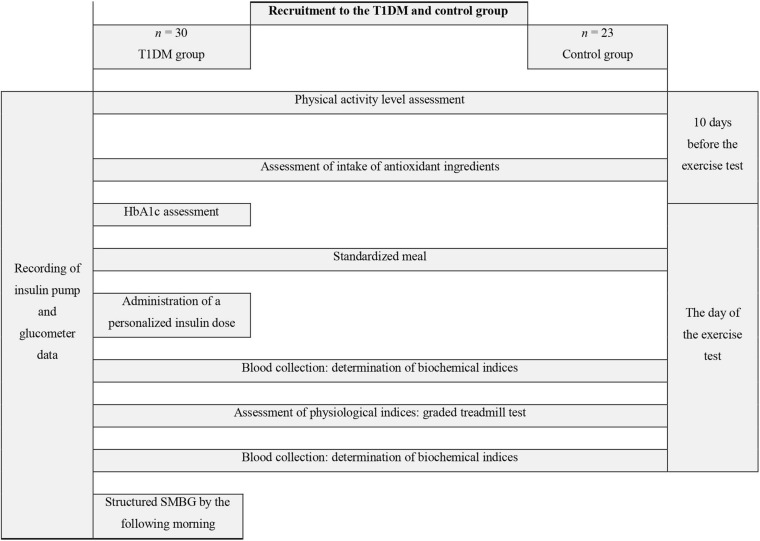
Study design diagram. T1DM, type 1 diabetes mellitus; HbA1c, glycated hemoglobin; SMBG, self-monitoring of blood glucose.

An overview of the study design is shown in Figure 1. Ten days prior to the commencement of physical capacity testing, the T1DM group males were given a continuous glucose monitoring system Dexcom G4 (display off) to wear until the start of the aerobic testing. The day before the exercise testing, each participant underwent fasting measurements of selected somatic indices. Body mass was determined by using Tanita BIA 547 scales, body height was measured with a SECA-210 stadiometer to the nearest 1 mm, and body composition was assessed with the bioelectric impedance method and an AKERN BIA 101 analyzer (CE0051 certificate, Council Directive 93/42/EEC concerning medical devices). Total body fat mass, lean body mass, total body water, extracellular water, and body cell mass were established.

To determine the percentage of HbA1c, the high-performance liquid chromatography method was applied in a hospital laboratory. Two hours before the physical capacity test and after the glycemia measurement, each patient consumed a standardized meal containing 50 g of digestible carbohydrates [3 carbohydrate exchangers: yogurt cereal (255 kcal) + 2 carbohydrate exchangers: bananas (116 kcal)]. Next, the participants took a dose of insulin calculated in accordance with their individual conversion rates and then reduced by 25% (Gawrecki, 2014; Picu et al., 2017). In addition, 30 min before exercise, the basal insulin infusion was reduced by 50% per hour.

In all participants, an approximate assessment of physical activity level was conducted on the basis of the daily number of steps, measured with a Garmin G5 device.

### Nutrition Assessment

In order to exclude nutrition as a factor potentially influencing the study results, a qualitative and quantitative evaluation of the diet was carried out. For this purpose, the participants kept dietary diaries for 7 days preceding the physiological and biochemical testing and completed a questionnaire on the frequency of selected food product consumption. Using the Diet 5.0 software (Food and Nutrition Institute, 2012, Warsaw, Poland), the dietary intake of energy, macronutrients (proteins, fats, carbohydrates), selected vitamins (C,E, and A), and the provitamin A (β-carotene) was estimated. Vitamin A, C, and E, as well as β-carotene were selected because they are involved in the non-enzymatic mechanisms counteracting oxidative adverse changes in the cell (Wang et al., 2012; Godala et al., 2017).

The frequency of consuming food products as a source of exogenous antioxidants was also verified with the use of a questionnaire. The current listing method was used for quantitative research, and a 7-point scale was applied to determine consumption rates, with the value of “1” corresponding to “never” and “7” standing for “several times a day” (Gronowska-Senger, 2013).

### Aerobic Capacity Level Test

The test was performed on a mechanical treadmill (Saturn 250/100R, h/p/cosmos, Germany). The exercise began with a 4-min warm-up at a speed of 7 km h^–1^, with an inclination angle of 1°. The running speed was then increased by a 1.0 km h^–1^ increment every 2 min to the point at which the participant was no longer willing to continue owing to exhaustion or reached a limit point at which an increase of running speed did not result in a rise in the continuously monitored indicators such as heart rate and VO_2_.

During the test, the peak oxygen uptake (VO_2_peak) and the second ventilatory threshold (VT2) were determined. Ergospirometry (Cortex MetaLyzer R3) was used to record the following indices: respiratory minute ventilation, the percentage of carbon dioxide in the exhaled air, minute oxygen uptake, minute carbon dioxide elimination, respiratory quotient, and respiratory equivalent for carbon dioxide. The heart rate during the test was measured with a sport tester (Polar Vantage V, Finland).

In order to determine VT2, changes in respiratory indices observed with increasing exercise intensity were analyzed. The criteria for the VT2 identification were as follows: the percentage of carbon dioxide in the exhaled air reached its maximal value and then decreased; the respiratory equivalent for carbon dioxide reached its minimum value and then increased; after exceeding VT2, there was a large non-linear increase in respiratory ventilation (Bhambhani and Singh, 1985; Binder et al., 2008). The VO_2_peak was considered to be the highest recorded value of this index.

The exercise-associated glycemia was controlled with a glucometer monitoring method (Contour Plus) (blood glucose checking: before a meal; before and immediately after the graded treadmill test; 30, 60, and 120 min after the test; and then, to minimize the risk of nighttime hypoglycemia, at 22:00, 24:00, and 3:00 the following morning) because continuous glucose monitoring system accuracy may be reduced after high-intensity exercise (Moser et al., 2016).

An hour after the aerobic capacity test, all participant data from the preceding 2 weeks (sports watch recordings, personal insulin pump data, and glucometer measurements) were downloaded with the use of dedicated software.

### Biochemical Evaluations

Lactate concentration (La^–^) was assessed in arterialized blood collected before the exercise test and at 3 and at 20 min after its completion. Lactate concentration was determined in plasma with the enzymatic method by using a Lactate PAP kit (BioMérieux, France) and a Spekol 11 spectrophotometer (Carl Zeiss Jena, Germany).

Before the aerobic capacity test and 60 min after its completion, in accordance with applicable standards, blood was collected from the cubital fossa vein (2 mL × 6 mL) to EDTA-containing tubes. The samples were then centrifuged at 2,000 rpm and the plasma was separated from the erythrocyte mass. The obtained plasma was stored in identical conditions, at −80°C, until biochemical analysis was performed. Biochemical analysis was conducted by using the same biochemical tests and equipment for all samples and following the reagent manufacturer’s instructions.

To assess the antioxidant status, an ImAnOX test kit was used (Immundiagnostik AG, Bensheim, Germany). The test directly measures the ratio of TAS to TAC of the tested material (test sensitivity: 130 μmol L^–1^, KC5200). In this test, antioxidants present in the sample react with a certain amount of hydrogen peroxide (H_2_O_2_), provided exogenously. The concentration of the residual peroxide is determined photometrically.

PerOx determination, being the ratio of TOS to the TOC of the serum, was also performed with the Immundiagnostik AG test kit (test sensitivity: 7 μmol L^–1^, KC5100; reference values: TOS/TOC < 180 μmol L^–1^—low peroxidized lipid concentration, 180–310 μmol L^–1^—average peroxidized lipid concentration, and TOS/TOC > 310 μmol L^–1^—high peroxidized lipid concentration), with the use of a calibrator provided by the manufacturer. In this test, the concentration of peroxidized lipids in the material was evaluated. Measurements were performed with a Chromate 4300 Microplate Reader (Awareness Technology, United States) at a wavelength of 450 nm with reference to the calibration plate.

The obtained values of optical density for individual samples were converted in accordance with the formulas indicated by the test manufacturer. The concentrations of TAS/TAC and TOS/TOC were determined in a single measurement. The oxidative stress index (OSI) was defined as the percentage ratio of TOS/TOC levels to TAS/TAC levels (TOS/TOC/TAS/TAC) expressed in arbitrary units (Erel, 2005).

Commercially available enzyme-linked immunosorbent assay (ELISA) system kits (Human Sandwich ELISA kit, SunRed Biotechnology Company, Shanghai, China) were used in accordance with the manufacturer’s recommendations to determine the serum VEGF (201-12-0081), 3-nitrotyrosine (201-12-1426), and endothelial nitric oxide synthase 3 (eNOS-3) (201-12-0920) concentrations.

### Statistical Analysis

The normality of continuous variable distribution was assessed using the Shapiro-Wilk test. Pearson correlations with Holm’s correction were used to evaluate the correlations.

Differences between the groups were analyzed with Student’s *t*-test or non-parametric tests (such as the Wilcoxon test), as appropriate. When comparisons were made in the same group (before vs. after the test), paired tests were applied.

Multiple regression analysis was used to determine the associations of the independent variables with the oxidative stress level, with respect to the potential confounding variable (the healthy/sick grouping variable; the percentage of fat mass; VO_2_peak level). Variables such as BMI, lean body mass, fat mass, total body water, and extracellular water volume were used for descriptive purposes only. The Pwr.t2n.test (two samples with unequal *n*) function served to perform a power calculation.

The study results are presented as arithmetic means ± standard deviations. All statistical analyses were performed with the R ver. 3.6.1 statistical software^1^. The results were considered significant at the significance level of *p* < 0.05.

## Results

### Somatic Indices Assessment and Clinical Characteristics of T1DM

A difference was revealed for the participants’ body fat mass percentage (*p* = 0.0268) together with a borderline discrepancy in age (*p* = 0.0441). However, no significant differences were observed between the groups for the majority of somatic indices (Table 2). The diabetic group was characterized by satisfactory glycemic control and moderate activity level on the basis of Tudor-Locke and Bassett classification using step counts (Bassett et al., 2017). Physical activity was not determined for the control group, although earlier control samples of students from the same population used in our previous research labeled this group as having a moderate or high level of physical activity (Gawrecki et al., 2012; Vlahek et al., 2013). Detailed clinical characteristics of the T1DM group are presented in Table 3.

**TABLE 2:** Somatic indices in the investigated groups.

Indices	T1DM group (mean ± SD)	Control group (mean ± SD)	*p*
Age (years)	23.4 ± 5.1	24.7 ± 2.9	0.0441*
Body height (cm)	179.0 ± 8.0	180.5 ± 6.4	0.4774
Body mass (kg)	78.3 ± 13.7	74.7 ± 8.6	0.2481
Body mass index (kg m^–2^)	24.3 ± 3.1	22.9 ± 2.1	0.0702
Lean body mass (kg)	64.2 ± 7.8	64.3 ± 6.9	0.9848
Fat mass (kg)	14.1 ± 7.5	10.5 ± 3.9	0.0898
Fat mass percentage (%)	17.1 ± 6.2	13.9 ± 4.2	0.0268*
Total body water (L)	47.0 ± 5.9	47.0 ± 5.2	0.9871
Total body water (%)	60.7 ± 4.1	61.0 ± 10.1	0.2290
Extracellular water (L)	19.7 ± 2.7	20.4 ± 5.4	0.8575

T1DM, type 1 diabetes mellitus.

*p < 0.05.

**TABLE 3:** Clinical characteristics and selected parameters of continuous glucose monitoring in T1DM patients.

Variables	Mean ± SD
Diabetes duration (years)	11.4 ± 6.0
Time of CSII use (years)	7.0 ± 4.1
HbA1c concentration (%)	7.2 ± 0.9
HbA1c concentration (mmol mol^–1^)	55
DDI (IU)	56.0 ± 15.8
DDI (IU kg^–1^)	0.72 ± 0.19
Number of steps per day (*n*)	8771.9 ± 2695

**CGM data**	

Mean glycemia from CGM (mg dL^–1^)	155 ± 34.9
Median glycemia from CGM (mg dL^–1^)	145.3 ± 36.6
CV from CGM (mg dL^–1^)	44.0 ± 6.1
Time spent below 70 mg dL^–1^ (%)	10.8 ± 8.0
Time spent below 54 mg dL^–1^ (%)	4.3 ± 4.3

*T1DM, type 1 diabetes mellitus; CSII, continuous subcutaneous insulin infusion (treatment with a personal insulin pump); HbA1c, glycated hemoglobin; DDI, daily dose of insulin; CGM, continuous glucose monitoring from 10 days before the test; CV, coefficient of variation (glycemic variability).*

### Nutrition Analysis

In the T1DM group, the energy supply from food was 2,314 ± 668 kcal/day, including 44.9 ± 9.1% of energy from carbohydrates, 39.4 ± 7.3% from fat, and 15.7 ± 4.4% from proteins. In the control group, the respective values equaled 2,522 ± 712 kcal/day, 47.1 ± 8.3%, 37.1 ± 8.8%, and 15.8 ± 5.2%.

The dietary supply of antioxidant vitamins was similar in T1DM patients and the control group: 88.4 ± 82.8 mg vs. 91.7 ± 89.9 mg for vitamin C, 11.3 ± 7.7 mg vs. 12.5 ± 9.8 mg for vitamin E, 1,001.3 ± 990.5 μg vs. 1,001.4 ± 712.0 μg for vitamin A, 2,760.1 ± 5475.2 μg vs. 2,831.4 ± 3983.0 μg for β-carotene, respectively.

The analysis of consumption frequency demonstrated that tomatoes, onions, lettuce, red peppers, carrots, butter, and black pepper were the most frequently consumed foods in both studied groups, with an average consumption rate of 3.9–4.4, indicating that the products were consumed 2–3 times a week on average. Other products rich in antioxidants were consumed at variable rates within the respective groups, with no significant differences.

### Physiological and Biochemical Indices Assessment

Table 4 presents the physiological indices characteristics at the VT2 and maximal levels for both groups, as well as mean values of lactate concentration. For most indices, significant differences were found. The mean peak and threshold minute oxygen uptake were significantly lower (*p* ≤ 0.001) in the T1DM group when compared with the control group. A similar difference was observed for the respiratory minute ventilation and for average speeds and distances. On the contrary, significantly higher values were noted in the T1DM group for heart rate at the maximal level (*p* = 0.001). Lactate concentrations at 3 and 20 min after the maximum-intensity exercise were significantly lower in the control group (Table 4).

**TABLE 4:** Physiological and biochemical indices.

Indices	T1DM group (mean ± SD)		Control group (mean ± SD)		*p*	
			
	Max	VT2	Max	VT2	Max	VT2
t (min)	16.8 ± 3.1	9.0 ± 2.5	18.1 ± 3.2	11.7 ± 3.3	0.132	0.001*
v (km h^–1^)	12.4 ± 1.5	8.5 ± 1.2	15.9 ± 2.2	12.4 ± 2.1	≤0.001*	≤0.001*
HR (beats min^–1^)	197.4 ± 7.5	168.9 ± 9.7	188.7 ± 12.3	168.8 ± 9.6	0.001*	0.988
VO_2_peak (L min^–1^)	3.4 ± 0.5	2.6 ± 0.5	4.2 ± 0.5	3.3 ± 0.4	≤0.001*	≤0.001*
VO_2_peak (mL kg^–1^ min^–1^)	44.7 ± 5.7	33.0 ± 6.1	56.0 ± 7.3	45.2 ± 6.1	≤0.001*	≤0.001*
Ve (L min^–1^)	128.8 ± 19.6	67.7 ± 12.6	149.2 ± 24.0	95.6 ± 23.7	0.001*	≤0.001*
%VO_2_peak (%)	73.6 ± 7.8		80.8 ± 5.7		≤0.001*	
%HRpeak (%)	85.6 ± 3.9		88.4 ± 3.70		0.01*	
Distance (m)	2,464 ± 692		3,742 ± 951		≤0.001*	
La^–^_0_	1.6 ± 0.2		1.6 ± 0.2		0.5911	
La^–^_3_	13.7 ± 3.1		11.4 ± 2.6		0.007*	
La^–^_20_	8.1 ± 2.6		5.3 ± 1.6		≤0.001*	

T1DM, type 1 diabetes mellitus; Max, maximal index level; VT2, second ventilatory threshold index level; t, working time in the graded treadmill test; v, speed in the graded treadmill test; HR, heart rate; VO_2_peak, maximal oxygen uptake: global (L min^–1^) and relative to body mass (mL kg^–1^ min^–1^); Ve, respiratory minute ventilation; La^–^_0_, baseline lactate concentration; La^–^_3_, lactate concentration 3 min after the graded treadmill test; La^–^_20_, lactate concentration 20 min after the graded treadmill test. *p < 0.05.

The HbA1c concentration was not proven to be a predictor of VO_2_peak in the T1DM group (in univariate linear regression at *p* > 0.05).

### Assessment of Oxidant-Antioxidant Balance Indices

The mean concentration of TOS/TOC before and after the exercise test was significantly higher in the T1DM group as compared with the control group (*p* ≤ 0.001). The value of OSI was significantly higher in the T1DM group both before and after the maximum-intensity exercise (Table 5).

**TABLE 5:** Oxidative and nitrosative stress and endothelial function indicators before and after maximum-intensity exercise.

Indices	T1DM group (mean ± SD)			Control group (mean ± SD)			*p* T1DM vs. control^	
					
	Before	After	*P*	Before	After	*p*	Before	After
TOS/TOC (μmol L^–1^)	321.5 ± 151	380.1 ± 153.5	≤0.0001*	164.1 ± 57	216.6 ± 74.9	0.0000	≤0.0001*	≤0.0001*
Δ TOS/TOC (%)	24.2 ± 31.7			34.9 ± 31.1			0.0311	
TAS/TAC (μmol L^–1^)	361.0 ± 22.2	327.0 ± 31.1	≤0.0001*	351.5 ± 26.4	360.7 ± 24.5	0.04	NS	≤0.0001*
Δ TAS/TAC (%)	9.4 ± 7.1			−2.8 ± 5.9			≤0.0001*	
OSI	0.9 ± 0.1	1.18 ± 0.5	≤0.0001*	0.47 ± 0.169	0.60 ± 0.20	0.000	≤0.0001*	≤0.0001*
Δ OSI	0.28 ± 0.24			0.13 ± 0.09			0.0127	
3NT (nM L^–1^)	281.4 ± 205.2	347.2 ± 268.4	≤0.0001*	125.3 ± 71.2	169.8 ± 91.1	≤0.0001*	0.001	0.010
Δ 3NT (nM L^–1^)	65.8 ± 88.5			44.5 ± 50.8			0.2569	
eNOS-3 (ng mL^–1^)	15.2 ± 16.6	18.1 ± 20.0	≤0.0001*	4.4 ± 2.2	5.4 ± 2.2	≤0.0001*	≤0.0001*	≤0.0001*
Δ eNOS-3 (ng mL^–1^)	2.9 ± 5.3			0.9 ± 0.8			0.0833	
VEGF (ng L^–1^)	422.4 ± 260.1	506.2 ± 344.1	≤0.0001*	308.4 ± 184.2	359.8 ± 187.5	≤0.0001*	0.1087	0.068
Δ VEGF (ng L^–1^)	83.8 ± 141.7			51.5 ± 34.2			0.3775	

T1DM, type 1 diabetes mellitus; TOS/TOC, total oxidant status; Δ, change; TAS/TAC, total antioxidant status; OSI, oxidative stress index; 3NT, 3-nitrotyrosine; eNOS-3, endothelial nitric oxide synthase 3; VEGF, vascular endothelial growth factor; NS, not significant. *Power of the test for each significant comparison between groups was > 98% (0.98).

Additionally, we built a multivariate regression model to control for possible confounders (percentage of fat mass and VO_2_peak). Even after a revised regression analysis, the previously significant comparison between groups remained significant.

There was a negative correlation between the rise in TOS/TOC after the maximum-intensity exercise and the length of time a participant was considered to be hypoglycemic with glucose levels < 54 mg dL^–1^ (*p* = 0.028; *r* = −0.40) and < 70 mg dL^–1^ (*p* = 0.034; *r* = −0.38), as well as a positive correlation, at a borderline significance, between Δ TOS/TOC and the time spent with continuous glucose monitoring result of > 140 mg dL^–1^ (*p* = 0.049; *r* = 0.36) and median glycemia (*p* = 0.05; *r* = 0.35) (Table 6).

**TABLE 6:** Pearson correlation results.

Indices	*p*	*r*
Δ TOS/TOC: median of CGM	0.054	0.35
Δ TOS/TOC: mean of CGM	0.090	0.31
Δ TOS/TOC: time spent < 54 mg dL^–1^ of CGM	0.028*	–0.40
Δ TOS/TOC: time spent < 70 mg dL^–1^ of CGM	0.034*	–0.38
Δ TOS/TOC: time spent > 140 mg dL^–1^ of CGM	0.049*	0.36
Δ OSI: median of CGM	0.016*	0.44
Δ OSI: mean of CGM	0.027*	0.40
Δ OSI: time spent < 54 mg dL^–1^ of CGM	0.019*	–0.43
Δ OSI: time spent < 70 mg dL^–1^ of CGM	0.022*	–0.41
Δ OSI: time spent > 140 mg dL^–1^ of CGM	0.026*	0.40
Δ OSI: time spent > 180 mg dL^–1^ of CGM	0.036	0.38
Δ 3NT: time spent < 54 mg dL^–1^ of CGM	0.010*	0.46
Δ 3NT: time spent < 70 mg dL^–1^ of CGM	0.025*	0.41

Δ, change; TOS/TOC, total oxidant status; CGM, continuous glucose monitoring; OSI, oxidative stress index; 3NT, 3-nitrotyrosine.

*p < 0.05.

Nitrosative stress indicators (3-nitrotyrosine and eNOS-3) exhibited significantly higher levels in the T1DM group as compared with the control group both before and after the exercise. The change in these indicators did not differ between the groups. VEGF level was similar both before and after the exercise in the studied groups (so was the change in this indicator) (Table 5).

## Discussion

Here, we report data on the aerobic capacity and changes in the oxidant-antioxidant balance during a maximum-intensity exercise in T1DM patients compared with healthy participants. Significant differences were determined for most measured physiological indices. Specifically, patients with T1DM had significantly lower levels of aerobic capacity than healthy participants of a similar age and BMI. A further novel finding is that the T1DM group was characterized by significantly higher TOS/TOC levels both before and after the maximum-intensity test; also, intra-group comparisons were significant. Moreover, the level of oxidative stress measured with OSI before exercise was significantly higher in the diabetic group compared with the healthy participants. Significant intergroup differences were observed in nitrosative stress after maximum-intensity exercise assessing aerobic capacity.

Several previous studies revealed that T1DM patients, even with a short duration of disease, tended to have significantly lower VO_2_peak values as compared with their healthy peers (Komatsu et al., 2005; Nadeau et al., 2010; Williams et al., 2011; Lukács et al., 2012; De Lima et al., 2017). Additionally, we observed a higher heart rate response to exercise in adults with T1DM, confirming a reduced cardiorespiratory fitness level compared with control participants, a phenomenon that was already reported earlier (Williams et al., 2011). Unlike these earlier projects, however, this study included patients with very good glycemic control and participants who were free from chronic diabetic complications. Of note, it also involved adult patients and a matched control group.

Several studies have shown that lower VO_2_peak values were associated with poor glycemic control (Baldi et al., 2010). This is in contrast to other investigations, as well as our observation (Stettler et al., 2006). One could speculate that the lack of this correlation was due to the fact that our cohort did not include patients with very high HbA1c levels, in whom the negative effects of metabolic decompensation on VO_2_peak could have been more easily seen. Specifically, it was observed that the achieved VO_2_peak was lower in patients with T1DM than in healthy controls when their HbA1c exceeded 64 mmol mol^–1^ (8%) and was comparable when HbA1c level was < 53 mmol mol^–1^ (7%) (Tagougui et al., 2015).

As physical activity is an integral element of T1DM therapy (Farinha et al., 2018), it is necessary to understand the impact of exercise on the oxidant-antioxidant balance in affected individuals, as well as the risk-benefit ratio. The baseline (pre-exercise) and post-exercise TOS/TOC values were significantly higher in the T1DM group than among the controls. Moreover, the maximum physical exercise during which RONS were generated resulted in a greater percentage shift regarding oxidant-antioxidant balance measured by the OSI stress level toward the oxidation reaction in the group of patients with T1DM compared with the group of healthy participants.

According to the guideline standards provided by the PerOx test (Immundiagnostik AG), the baseline and post-exercise values should be classified as a high TOS/TOC concentration. In the control group, the concentration of TOS/TOC was low before the exercise and medium after the maximum-intensity exercise. This is the first such observation in T1DM, although there was one previous study showing similar results in type 2 diabetes patients (Picu et al., 2017). The possible reasons for this difference warrant some discussion. First, diabetes mellitus itself is known to create a pro-inflammatory and pro-oxidative milieu (Picu et al., 2017). Numerous studies imply that diabetes is accompanied by systemic oxidative and nitrosative stress, which results in the reduced activity of antioxidant enzymes, low levels of non-enzymatic antioxidants, and an increase in free radicals and pro-oxidants (Bigagli and Lodovici, 2019; González-Bartholin et al., 2019).

Physical exercise itself is a source of free radicals in the body. One source of RONS generated during vigorous physical activity is the increase in the rate of changes in the enzymatic respiratory chain inside the mitochondria of muscle cells (Gomez-Cabrera et al., 2008). RONS levels will rise as a result of blood redistribution during and with the cessation of physical activity. Intensified physical activity changes redox homeostasis in virtually all organs, tissues, and body fluids. Therefore, the source of the greatest increase in the number of free radicals and their reaction products in the blood does not always have to be the skeletal muscle (Nikolaidis et al., 2012). Damaged muscle fibers are another source of increased amounts of reactive oxygen and nitrogen species caused by exercise, especially following exercise involving eccentric contractions. Another possibility of free radical formation during exercise is the autoxidation of heme proteins: oxyhemoglobin and oxymyoglobin (Cooper et al., 2002). Other causes of RONS production during exercise include the autoxidation of catecholamines, which are produced in significant amounts during exercise, and an increase in internal body temperature, which, among others, lowers the activity of mitochondrial uncoupling proteins, which normally prevent excessive build-up of membrane potential owing to the H + gradient in mitochondria (Belviranlı and Gökbel, 2006).

Another factor that should be mentioned is the overall body composition, which was different between the study groups (Furukawa et al., 2004). Even after controlling for this variable and VO_2_peak, the observed difference in oxidant balance between the groups remained significant.

Antioxidant potential, which consists of enzymatic and non-enzymatic antioxidants, is largely determined by nutrition. This refers to the type and quality of food, as well as the proper distribution of energy from proteins, fat, and carbohydrates, as well as the correct supply of regulatory ingredients. In our research, nutritional analysis in both groups showed no deficiencies in terms of energy or quality, including antioxidant vitamins, in the consumed food products. This could not have had impact on the pre-exercise plasma antioxidant concentration in the participants, monitored as TAS/TAC.

The concentration of TAS/TAC in the group of diabetics decreased after exercise, which may indicate that non-enzymatic antioxidants participated in the elimination of RONS generated during muscle contraction. In the group of healthy men, clinically significant changes were not observed.

A significant difference in TAS/TAC levels between the groups after the graded treadmill test was demonstrated in our study. Interestingly, lower plasma concentrations of magnesium and zinc were recently observed in children with T1DM than in control groups. Zinc and magnesium play an important role in glucose metabolism and antioxidant response (Salmonowicz et al., 2014; Marjanac et al., 2020). Lower antioxidant status was also revealed in young Portuguese diabetic children (Castro-Correia et al., 2017). The decreased TAS levels noted in T1DM patients may impair the effectiveness of non-enzymatic antioxidant systems (Salmonowicz et al., 2014). Similar results were obtained in T1DM patients with short disease duration as compared with healthy individuals, as the diabetic group exhibited an increase in RONS generation, while the plasma antioxidant status remained unaltered (Reis et al., 2008). One could speculate that the lack of difference in TAS/TAC levels before the test was due to the fact that our cohort did not include patients with very high HbA1c levels, e.g., zinc deficiency was more pronounced in poorly controlled T1DM participants (> 9% HbA1c), and this correlated with the severity of hyperglycemia (Salmonowicz et al., 2014).

We found a significant correlation between the hypoglycemia and hyperglycemia episodes monitored within 10 days before the exercise test and the TOS/TOC increase after exercise (Table 6). No correlation was observed with TOS/TOC values before or after exercise. It is unclear why dysglycemia correlates only with an increase in oxidative stress but not with the baseline values. One explanation may be that baseline stress values are related with glucose markers only within periods longer than 10 days and/or correlate with more severe hyperglycemia and/or stress response is more sensitive to current glucose control. We believe that the greater TOS/TOC percentage variation in the control group could be due to the fact that the exercise test in this group lasted longer than in the T1DM group. The physical effort performed by healthy men during the exercise test resulted in a greater production of free radicals. This could have caused a greater lipid peroxidation, which was measured by TOS/TOC.

We have shown higher values of nitrosative stress indicators in the T1DM group as compared with the control group both before and after the exercise. Reactive nitrogen species act as important mediators in cellular physiology and are produced in numerous biochemical processes (Ishimoto et al., 2018). Under physiological conditions, nitric oxide is synthesized from L-arginine in a reaction catalyzed by eNOS. Prolonged high concentrations of nitric oxide can cause irreversible damage to cell membranes, proteins, mitochondria, endoplasmic reticula, and nucleic acids, as well as impair the function of many enzymes (Pérez-Torres et al., 2020). Nitrosative stress is associated with several cardiometabolic diseases, including arteriosclerosis, arterial hypertension, endothelial dysfunction, and diabetes (Pérez-Torres et al., 2020). In the present study, the eNOS-3 protein levels were significantly higher after maximum-intensity exercise in both groups (T1DM: 15.2 ± 16.6 ng mL^–1^ before exercise and 18.1 ± 20.0 ng mL^–1^ after exercise, *p* ≤ 0.0001; control group: 4.4 ± 2.2 ng mL^–1^ before exercise and 5.4 ± 2.2 ng mL^–1^ after exercise, *p* ≤ 0.0001). This is consistent with previous studies (McAllister et al., 2008). However, the Δ eNOS-3 value of 2.9 ± 5.3 ng mL^–1^ vs. 0.9 ± 0.8 ng mL^–1^ (*p* = 0.0833) did not differentiate between the groups.

Hyperglycemia is a significant factor inducing the formation of reactive nitrogen species. Nitrosative stress associated with diabetes (Pérez-Torres et al., 2020) may cause hemodynamic changes through overexpression of inducible nitric oxide synthase, which has been linked to vascular, cardiac, and renal tissue damage in streptozotocin-exposed rats (Wilcox and Gutterman, 2005). In diabetes, increased peroxynitrite production not only causes nitrosative stress, but also reduces the bioavailability of functional nitric oxide and contributes to impaired endothelial relaxation (Cheng and Pang, 2004). Hyperglycemia, one of the main symptoms of diabetes, through a series of reactions activates protein kinase C and induces the formation of advanced glycation end-products that are responsible for many of the complications occurring in this disease. Accelerated non-enzymatic formation of advanced glycation end-products in the presence of hyperglycemia increases the expression and activity of nicotinamide adenine dinucleotide phosphate oxidase, which subsequently produces more O^2–^ *via* protein kinase C activation. This is followed by elevated O^2–^ and unbound eNOS levels and increased inducible nitric oxide synthase activity, with a consequent rise in 3-nitrotyrosine levels (Guzik et al., 2000). Increased 3-nitrotyrosine concentration is a critical signaling marker that indicates progression of inflammatory disorders (Attia and Al-Radadi, 2016). Diabetic patients exhibit high plasma levels of 3-nitrotyrosine, which are associated with myocytes, fibroblasts, and endothelial cell apoptosis. Increased microvascular immunoreactivity of 3-nitrotyrosine correlates with glycemic control and levels of intracellular and vascular adhesion molecules (Pop-Busui et al., 2009).

Excess nitric oxide is oxidized to peroxynitrite, which initiates multiple pathological signaling events, including the elevation of VEGF concentration. Endothelial dysfunction is the major cause of vascular complications in diabetes. Under conditions of hyperglycemia and/or hyperlipidemia, increased production of RONS is observed, with concomitant reduced capacity of antioxidant systems (Khodadadi et al., 2018; Zhao and Singh, 2018; Shye et al., 2020). Endothelial cells produce many substances that are responsible for maintaining vascular homeostasis and thus influence the whole body condition. Oxidative and nitrosative stress leads to endothelial dysfunction, increasing vascular tension (Schalkwijk and Stehouwer, 2005). In diabetic patients, endothelial dysfunction constitutes an early and crucial step in the development of diabetes-associated vascular disorders and also contributes to the exacerbation of atherosclerotic changes (Sena et al., 2013). In many cases, endothelial dysfunction associated with diabetes may result from an impaired response of this tissue to insulin; therefore, endothelium becomes one of the therapeutic targets in diabetes (Ciulla et al., 2018). In our study, we observed significant changes in VEGF levels measured before and after maximum-intensity exercise (T1DM group: 422.4 ± 260.1 vs. 506.2 ± 344.1 ng L^–1^, *p* ≤ 0.0001; control group: 308.4 ± 184.2 vs. 359.8 ± 187.5 ng L^–1^, *p* ≤ 0.0001). However, we observed no significant intergroup changes in Δ VEGF (83.8 ± 141.7 ng L^–1^ in the T1DM group vs. 51.5 ± 34.2 ng L^–1^ in the control group; *p* = 0.3775). In a study by Hoier et al. (2013), conducted among nine young and healthy men, intense intermittent exercise provided a weak stimulus for VEGF secretion and endothelial cell proliferation, and intense intermittent training did not induce a sufficient angiogenic stimulus.

Undoubtedly, oxidative and nitrosative stress is one of the mechanisms affecting complications in patients with T1DM. Therefore, the direction of future research should be extended to the use of physiological and biochemical indices obtained during a graded test to prepare individual plans for systematic physical activity, and direction and magnitude assessment of pro-oxidant-antioxidant balance shifts. Regular physical activity promotion should be enhanced, with special consideration of the barriers specific to T1DM patients, with a view to lowering BMI and improving body composition, both of which are beneficial to overall health (Brazeau et al., 2012).

Finally, some limitations of the study should be acknowledged. Firstly, in this kind of clinical research, there are some factors potentially influencing the study results which remain beyond the scope of the project. It would have been ideal to have corresponding physical exertion parameters from our control group sample as well but owing to a lack of sufficient funding, we were unable to gather data from these participants. For the same reason, we were unable to assess the plasma levels of some antioxidants (e.g., zinc, magnesium). We also did not include women in this study because, in accordance with our previous experience, female T1DM patients tend to be much less willing to participate in research involving very intensive exercise. Thus, the study results cannot be generalized to women. Furthermore, we did not use any of the earlier proposed methods to define the evaluated maximum oxygen capacity (Tarnus et al., 2011). Additionally, the indices used to evaluate the degree of damage to DNA and muscle cells were not determined. With high oxidative stress, inflammation occurs in the muscle cells, which causes their damage. Fat percentage difference between the groups might also have influenced our results. Patients with higher fat percentage worked harder during the exercise test: that is why they could have developed higher stress oxidative parameters than the slimmer participants in the control group. Finally, the food frequency questionnaire method may inaccurately assess nutrient intakes, which could have potentially affected the study results. Nevertheless, this tool is still commonly used in scientific research owing to its convenience, low cost, and a user-friendly interface.

## Conclusion

The treadmill test performed until exhaustion showed that aerobic capacity was significantly lower in young T1DM patients with glycemic control than in young healthy individuals. The treadmill test turned out a considerable oxidative and nitrosative stress among diabetics, which could have caused increased inflammation in this group as compared with healthy participants’ response to such effort. A large increase in VEGF levels in diabetics after exercise until exhaustion may put them at risk owing to the activation of retinopathy.

Recently, there have been more and more young people with T1DM who participate in sports both professionally and recreationally. In our study, we wanted to present the risk that diabetics faced during single maximal effort until exhaustion, which is usually applied to determine the effort capability of athletes who start their training cycles. Physical training in which repeated physical efforts of a certain VO_2_max intensity are implemented is a form of hormesis against which the body mobilizes and activates many systems and metabolic reactions that exert a positive impact on the body.

Our study can help inform coaches and sports physicians caring for athletes with T1DM that in these cases, excessive load during exercise can constitute a health risk. Further research seems to be necessary both in the evaluation of different types of efforts, including those with predominant eccentric contractions, and in the assessment of systematic training implemented in different forms.

## Data Availability Statement

The original contributions presented in the study are included in the article/supplementary material, further inquiries can be directed to the corresponding author/s.

## Ethics Statement

The studies involving human participants were reviewed and approved by Jagiellonian University Bioethics Committee (approval No. 1072.6120.113.2017). The patients/participants provided their written informed consent to participate in this study.

## Author Contributions

ŁT, BM, and WP contributed to the conception and design of the study. ŁT and BM organized the database. BM performed the statistical analysis. ŁT, BM, MM-T, WP, SM, and TP wrote the first draft of the manuscript. ŁT, BM, MTM, and WP wrote the sections of the manuscript. All authors contributed to manuscript revision, read, and approved the submitted version.

## Conflict of Interest

The authors declare that the research was conducted in the absence of any commercial or financial relationships that could be construed as a potential conflict of interest.

## Publisher’s Note

All claims expressed in this article are solely those of the authors and do not necessarily represent those of their affiliated organizations, or those of the publisher, the editors and the reviewers. Any product that may be evaluated in this article, or claim that may be made by its manufacturer, is not guaranteed or endorsed by the publisher.

## Footnotes

1
http://www.r-project.org/
